# Understanding How Adolescents Think about the HPV Vaccine

**DOI:** 10.3390/vaccines8040693

**Published:** 2020-11-18

**Authors:** Robyn A. Pennella, Katherine A. Ayers, Heather M. Brandt

**Affiliations:** St. Jude Children’s Research Hospital, 262 Danny Thomas Pl, Memphis, TN 38105, USA; Robyn.Pennella@stjude.org (R.A.P.); heather.brandt@stjude.org (H.M.B.)

**Keywords:** human papillomavirus, human papillomavirus vaccine, human papillomavirus vaccination, cancer prevention, adolescents

## Abstract

Despite educational efforts, Tennessee human papillomavirus (HPV) vaccination rates are 43%, among the lowest in the United States. This study examined how adolescents think about the HPV vaccine to identify patterns and misconceptions to enhance educational efforts. Adolescents (ages 11–12) (*N* = 168) responded to open-ended questions regarding their thinking about the HPV vaccine. Data were analyzed and interpreted using qualitative thematic analysis. Three domains of themes emerged from responses: (1) characteristics of HPV vaccination, (2) knowledge-related themes, and (3) beliefs-related themes. Prevention of HPV and cancer was the most referenced characteristic of HPV vaccination followed by HPV vaccine rates and HPV vaccine efficacy. Student inquiries were mostly centered on HPV vaccine composition, administration, duration and how the vaccine interacts with the body. Some responses indicated a desire for more information about HPV not specific to the HPV vaccine. Overall, adolescent attitudes were positive towards the HPV vaccine. This study highlights specific questions adolescents have about the vaccine that can be used to tailor future HPV educational efforts, empowering adolescents with the knowledge to be more active students in the decision-making process. In addition, the potential for adolescents to serve as community advocates for the vaccine should be considered for future interventions.

## 1. Introduction

Each year in the United States (U.S.), 34,800 women and men are estimated to be diagnosed with a cancer attributed to human papillomavirus (HPV) infection [1]. HPV vaccination could prevent more than 90% of these cancers [1]. Uptake of HPV vaccination in the U.S. has however been slow. Based on the most recent data, 54% of adolescents age 13–17 have completed the HPV vaccine series with completion rates in Tennessee being much lower at 43%, among the lowest in the country [2,3,4]. General recommendations are that children receive the vaccination at 11–12 years of age and as early as age 9 [5,6,7,8]. There is an urgent need for effective interventions to increase uptake of HPV vaccination to *Healthy People 2020* levels of 80% and prevent HPV-associated cancers [9,10].

Educational efforts to improve HPV vaccination rates have historically targeted healthcare providers and parents, not adolescents [11,12,13,14,15,16,17]. Adolescents, therefore, have been largely marginalized from the decision-making processes associated with HPV vaccination [17,18,19,20]. One study found that adolescents felt like passive students in the HPV vaccine decision-making process even when parents and clinicians felt that they were active students [21]. Research suggests that many adolescents want more information to improve their knowledge and awareness of the HPV vaccine and want to be a part of [20,22,23,24] and feel they should be [25] a part of the decision-making process. These findings highlight the need to develop “appropriately tailored interventions addressing the adolescents self-identified desire to be better informed” [20].

Tailoring health communication aims to enhance the relevance of the information presented in order to produce greater desired changes in response to the communication [26,27]. This, of course, implies that we know enough about the target audience to understand what is relevant from their perspective. Tailoring HPV vaccination interventions for adolescents, however, is challenging as very little is known about how adolescents think about the vaccine. Studies that do explore attitudes towards the HPV vaccine in adolescents are often limited to adolescents who have received the vaccine, excluding perceptions of those who are vaccine hesitant [13,21,22,28]. A recent study exploring HPV vaccine acceptance in African American mothers and their adolescent daughters (both vaccinated and unvaccinated) revealed that positive attitudes towards the HPV vaccine stemmed from viewing the vaccine as a cancer-prevention measure, concluding that tailoring HPV vaccine education with a cancer prevention message may appeal to adolescents [29]. Across all three studies, adolescents expressed a need for more information about HPV and HPV vaccination safety, side effects, and effectiveness, regardless of their vaccination status, identifying the need for better HPV-related education targeting adolescents.

St. Jude Children’s Research Hospital formed a school-based outreach program, the St. Jude Cancer Education and Outreach (SJCEO) program, in 2006 that utilizes education to promote healthy lifestyle choices with the aim of reducing a child’s lifetime risk of cancer. The outreach curriculum encourages visible thinking in students by using “thinking routines” [30] throughout the curriculum to critically engage K-12 students in conversations around complex issues related to cancer risk. In 2018, SJCEO conducted a five-day cancer educational program during the Shelby County School (SCS) district Summer Superintendent’s Learning Academy (SSLA). SSLA was a free, five-week summer program offered through the SCS district available to all current SCS students on a first-come-first-serve basis. The outreach program was designed to encourage students to think critically about topics related to cancer formation, treatment, and prevention, specifically highlighting HPV and the HPV vaccination in the greater context of cancer prevention. In this study, we described the thinking patterns observed from adolescents ages 11–12 when engaged in conversation around the HPV vaccination as a cancer preventive practice.

## 2. Materials and Methods

The study was a secondary data analysis of student artifacts collected as part of the SSLA program. We used a qualitative design to analyze existing data collected in 12 SCS during the 2018 SSLA and aimed at understanding how adolescents (ages 11–12) (*N* = 168) think about the HPV vaccine. The SCS district is a larger urban school district in the Memphis, Tennessee-area, serving over 100,000 students who are predominantly people of color (73% African American; 15% Hispanic/Latinix; 56% economically disadvantaged). No individual-level sociodemographic data were collected for the students. St. Jude’s Institutional Review Board reviewed study information and approved it as exempt under exemption 4, involving existing data in which subjects cannot be identified.

Data analysis focused on the fourth day’s lesson dedicated to carcinogens and viruses. During this lesson, students were introduced to HPV, namely what it is, how it spreads, and how it potentially leads to cancer. This was followed by an introduction to and discussion of vaccines, calling attention to the HPV vaccine. Once students received information about HPV and the HPV vaccine, they were asked to respond to four questions on HPV and HPV vaccination. Specifically, students were asked: (1) What excites you about the HPV vaccine? What’s the upside? (2) What worries you about the HPV vaccine? What’s the downside? (3) What do you still need to know about the HPV vaccine? and (4) What is your stance or opinion on the HPV vaccine?. Students wrote down their individual responses in their workbooks. Written responses were transcribed into Microsoft Excel and then subsequently uploaded to MAXQDA, a standard software for coding and analyzing qualitative data, to manage and code students’ responses to each of the four questions using established approaches for thematic coding. The constant comparative method was used to identify emerging themes comparing information in each subsequent workbook to previously reviewed workbook data.

## 3. Results

Across all four questions, there were 168 total student responses with specific responses per question reported here. Three major domains of themes emerged from student responses: (1) characteristics of HPV vaccination; (2) knowledge-related themes; and (3) beliefs-related themes. See Table 1 for a description of emergent themes.

### 3.1. Characteristics of HPV Vaccination

Specific characteristics of HPV vaccination were discussed in the students’ responses. In some instances, the responses demonstrated knowledge of HPV vaccination or reaffirmed belief in the value of HPV vaccination.

#### 3.1.1. HPV Vaccination is Prevention

One hundred twenty-six students indicated that the HPV vaccination was a prevention measure. Eighty-seven students spoke specifically of prevention of HPV transmission. For example, student WH012 expressed, “I find that HPV can spread very easily and fast that is why we need HPV vaccine.” Similarly, student CR010 stated that, “It spreads to anyone who doesn’t have the vaccine.” Thirty-three students spoke of prevention in general terms. For example, student HO011 responded that “it protects you.” A small subset of students explicitly mentioned the HPV vaccine as a cancer-prevention measure. For example, student LW008 stated, “The most exciting thing to me is that this HPV vaccine could prevent cancer and help build our immune system.”

#### 3.1.2. HPV Vaccination Rates

Forty student responses were related to HPV vaccination rates. Twenty-four student responses explicitly stated whether they would get the HPV vaccine. There were more responses that expressed they would get the HPV vaccine (*N* = 18) than those that expressed they would not (*N* = 6). For example, student LW012 expressed, “I would want to get it” while student PE006 stated, “I wouldn’t try it at all and it will kill me.” Thirteen student responses underscored the relationship between low HPV vaccination rates and persistent spread of HPV. For instance, student BR008 stated that, “If people don’t have a vaccine shot they can have the HPV get spreaded [*sic*].” Similarly, student AC003 mentioned that “I find that HPV can spread very easily and fast that is why we need HPV vaccine.” Two student responses pointed toward barriers to HPV vaccination. Student DE010 stated “If your busy and really wanna [*sic*] get a vaccine you won’t have time to get a shot” while student WS006 highlighted that, “Everyone may not be able to get one.” Student BR010 took on the role of advocate when they responded “Parents you need to let your children get a vaccine shot because it’s not bad”.

#### 3.1.3. HPV Vaccine Efficacy

Thirty-eight student responses reflected inquires and concerns around the effectiveness of the HPV vaccine. Student CR008 asked “What if it doesn’t work?” Similarly, student HO014 asked, “Will it work?” while student LW006 claimed, “The vaccine might not work and I might not be responded [*sic*] to it.”

### 3.2. Knowledge Related Themes

Knowledge-related themes and subthemes emerged from the students’ responses across the questions. Students expressed a desire to learn more about HPV and HPV vaccination. Two themes emerged related to desired knowledge: (1) desired HPV vaccination knowledge and (2) desired HPV knowledge. Desired HPV vaccination knowledge was organized into four subthemes to further elucidate specific types of HPV vaccine knowledge wanted by the students.

#### 3.2.1. Desired HPV Vaccination Knowledge

Ninety-seven student responses indicated a desire for more HPV vaccination knowledge. Forty-six responses indicated a desire to know more about the HPV vaccine administration process. Student DT003 asked, “Can our parents be in there with us?” while student LW011 stated, “The downside is you could get scared because you will be like wonder if its hurts wonder if the needle is sharp.” Thirty-three student responses indicated a desire to know more about HPV composition. For example, student HO015 asked, “What are all the ingredients in the vaccine?” Student DE005 similarly asked, “What kind of chemicals are in the vaccine?” Twelve student responses indicated a desire to know more about how the vaccine interacts with the body upon administration. Student SD012 claimed “I need to know about the vaccine and what it will do to me.” Student CR001 asked, “How does it work?” Six student responses indicated a desire to know more about HPV vaccine durability. Student PE007 argued that “You should know if you get the shot how long will it last” while student WS005 asked, “Is it going to last for a lifetime or a month?”

#### 3.2.2. Desired HPV Knowledge

Forty-five student responses indicated a desire to gain more information related to HPV overall and not specific to HPV vaccination. For example, student WH009 asked, “What, why, how do you have HPV?” Student RC002 asked “How does it spread to [*sic*] skin to skin contact?” Similarly, student CR004 stated, “I want to find out how HPV get in your body and why so many people get it?”

### 3.3. Beliefs Related Themes

In addition to desired knowledge, students expressed beliefs about HPV vaccination that were both positive and negative.

#### 3.3.1. Positive Perceptions of the HPV Vaccine

One hundred forty student responses described the nature of students’ overall orientation towards the HPV Vaccine. One hundred twenty student responses indicated that there was no concern around the HPV vaccine and that the HPV vaccine is good and helpful. Student BR004 explicitly claimed that “it’s helpful.” Student BR006 argued that “It’s great and doctors should continue to develop it,” and student RC015 stated that, “They are good so there are no worrisomes [*sic*].”

#### 3.3.2. Negative Perceptions of the HPV Vaccine

Twenty student responses indicated negative perceptions of the HPV vaccine. Student LW012 expressed “I think its just a real virus that’s being injected into you.” Student WH010 claimed “It is dangerously bad and deadly.”

## 4. Discussion

The results of this qualitative study indicated adolescents were capable of engaging in complex thinking about HPV and the HPV vaccine. Students were able to comprehend basic information about HPV, such as HPV can cause cancers to form in the body and getting the HPV vaccine reduces the spread of HPV in the general population while also minimizing their own risk of developing an HPV related cancer. In this way, they were able to understand they are not only protecting themselves when they are vaccinated, but also protecting members of their community. The findings suggest adolescents may be motivated by educational efforts that facilitate deep thinking around the population implications of the HPV vaccine and the repercussions that choosing to vaccinate or not to vaccinate might have on their community. It is interesting to note, however, that students expressed this understanding as either an excitement or a worry depending on whether they focused their thinking on individuals who choose to vaccinate or individuals who choose *not* to vaccinate. For instance, BR003 expressed excitement that “you can get vaccinated and if someone is infected than you won’t get infected” while BR008 expresses worry that “if people don’t have a vaccine shot they can have the HPV get spreaded [*sic*].” indicating that students were implicitly aware of the individual’s right to choose whether or not to vaccinate and that many individuals may choose not to vaccinate. One approach that future educational efforts might take is to explicitly highlight this dilemma by facilitating conversations around reasons why people choose either to vaccinate or not. More research, however, is needed to determine the impact this would have on adolescents’ perceptions of the HPV vaccine—whether it fuels adolescents’ excitement or exacerbates adolescents’ worries and how either outcome influences adolescents’ stance on whether or not to self-vaccinate.

Students also expressed many worries about the vaccine that appear to be driven by unanswered questions. While many of these same worries have been identified in previous studies [13,28,29], these worries are often expressed in interviews after the HPV vaccination series has begun or been completed. This is important for contextualizing the HPV vaccine conversation with adolescents as research shows adolescents are largely left out of the HPV conversation during the decision-making process in the doctor’s office [17,18,19,20,23,24,25]. This study engaged adolescents in the classroom setting at a local elementary school. The classroom, as opposed to the pediatrician’s office, is a space where adolescents are expected to actively engage with new material by asking questions and sharing insights, especially in the context of science education where adolescents are frequently asked to “think like scientists” by approaching new ideas with a critical skepticism. The pediatrician’s office, on the other hand, can often create a culture of stress and anxiety in adolescents, which then contributes to their marginalization during the decision-making process [20]. Hosting our health education initiative in a classroom as opposed to a pediatrician’s office allows adolescents to actively participate in conversations around their health without the stress of being in a pediatrician’s office. This can better prepare them for these patient-pediatrician conversations around the HPV vaccine as they approach the age of HPV vaccine eligibility. The classroom provides a safe practice space for adolescents to field their questions around HPV vaccine. Future educational efforts should consider partnering with science educators to develop HPV-related curriculum to be used in the formal science classroom that addresses these worries that adolescents might have, specifically where the vaccine came from, who invented it, and how do we know it works. This can be done using inquiry-based learning as described by the National Research Council’s 3-Dimensional Learning Framework [31]. At the same time, interventions designed to enhance HPV vaccination uptake should consider working with pediatricians and nurses to foster an inclusive environment that welcomes adolescents to more actively participate in the decision-making process. More research is needed to determine the potential impacts of this type of multi-pronged approach towards promoting HPV vaccination.

Our data also revealed a tendency for adolescents to take on the role of “advocate.” Students often made declarative stances that indicated a need to advocate for the HPV vaccine to parents and other adolescents, revealing that adolescents are aware of controversies related to either HPV vaccination specifically or vaccinations more generally. It is unclear as to whether this push towards advocacy is driven by student excitements about the preventative power of the HPV vaccine or student worries about the consequences of vaccine refusers. More research is needed to better understand these driving forces and the potential for training adolescents to advocate for HPV vaccination during their own vaccination decision-making process.

In a similar line of thinking, students expressed awareness of structural barriers that limit vaccination access. This may be a result of a large portion of our sample coming from underserved communities where they are more likely to be exposed to the effects of structural violence from an early age. For instance, when WS006 pointed out that “everyone may not be able to get one,” they underscore these structural barriers to vaccine access that carry implications not only for an individual but also for an entire community. This, coupled with the tendency of students to take on the role of advocate, suggests that adolescents from underrepresented populations in particular may respond well to framing HPV conversations through a social justice lens. Future educational efforts should intentionally design programs that acknowledge and address structural barriers to vaccination access for adolescents regardless of socioeconomic status and highlight programs, such as Vaccines for All, that aim to minimize these barriers. By doing this, these future efforts can serve as an introduction to the relationship between healthcare and policy while also introducing the HPV vaccine to adolescents.

While our study contributes to the overall understanding on how early adolescents think about the HPV vaccine, it is clear that students still desire more information about the vaccine. Negative stances towards the HPV vaccine stem from common vaccine misconceptions. Future educational efforts should focus on instilling a deeper and fuller understanding of the HPV vaccine specifically and vaccines in general in this population. Our results suggest that adolescents are eager to learn more about their health. This has significant implications for the potential role of adolescents in the vaccine decision-making process. Pediatricians may find success in patient uptake of the HPV vaccine by explicitly framing the HPV vaccine as a cancer-fighting measure during their patient-clinician and parent-clinician conversations while also paying special attention to structural barriers that may plague their patient and the patient’s family. Seeing as the adolescents in our study responded well to framing the HPV vaccine as a cancer-prevention measure, future studies should explore how parents of HPV vaccine eligible adolescents respond to this same framing. By providing adolescents with an opportunity to critically think about the HPV vaccine in a classroom setting we are equipping them with knowledge that can be used to better participate in their own medical decision-making processes while also gaining valuable insight into the ways they perceive the vaccine, which can then be used to better tailor future interventions.

### Limitations

While our study is one of the few focused on understanding the thinking of adolescents related to HPV vaccination, there were several limitations to note. Working with adolescents had some challenges especially when collecting written response data. Responses were sometimes illegible to the researchers or off-topic. Researchers conducting similar studies should consider coupling our method with student interviews to resolve confusion that may arise from solely using written responses or including closed-ended options to gather information from the adolescent perspective. Four questions were used to ask about the HPV vaccine, but many student responses were regarding HPV itself. This discrepancy may have been a result of facilitators failing to emphasize this distinction and/or the wording of our questions. Further, because the data in our study were a part of a larger cancer educational intervention, this study was not designed with analysis in mind, rather it is a by-product of these cancer educational activities and emergence of insight into adolescent understanding of HPV vaccination. Our study did not consider descriptive characteristics, such as social and demographic factors, when exploring adolescent understanding of the HPV vaccine. For example, sex or gender may matter and is unknown for students in our study. There has been research to suggest that females hear about the vaccine from a much younger age than their male counterparts, and this can contribute to the lower vaccination rates seen in males [20]. While this does weaken the analysis in terms of our inability to assess gender differences, we feel that, given what we do know about the student demographics served by SCS (72% Black; 15% Hispanic; 56% economically disadvantaged), these data provide valuable insights around how adolescents from underserved communities, particularly Black and Brown adolescents from low SES backgrounds, think about HPV and the HPV vaccination. Future research should collect information on characteristics of adolescents to better understand possible differences and thus, inform tailored educational efforts.

## 5. Conclusions

In conclusion, adolescents (ages 11–12) are capable of complex thinking around HPV and HPV vaccination and express a desire for more knowledge on a variety of topics related to the vaccine, including vaccine composition, the body’s response to the vaccine, and immunization duration.

Although this was a small study, our results make notable contributions to the field as a gap in the literature exists around adolescent thought patterns regarding HPV and the HPV vaccine. Understanding how adolescents think about the HPV vaccine helps researchers to better tailor future efforts. Specific worries and concerns identified in this study should be incorporated into future health educational efforts to provide adolescents with a strong enough knowledge base to become more active participants in their medical decision-making processes.

## Figures and Tables

**Table 1:** Description of emergent themes.

Theme	Definition	Sub-Theme	Definition	Example
Characteristics of HPV Vaccination				
HPV Vaccination is prevention		Prevention of HPV transmission	This subtheme reflects students connection between the HPV vaccine and the spread of HPV.	“It excites me that HPV vaccine is here to help people who can catch HPV”
				“You can get vaccinated and if someone is infected than you won’t get infected”
				“It spreads to anyone who doesn’t have the vaccine”
				“If people don’t have a vaccine shot they can have the HPV get spreaded” [*sic*]
				“I would be
				worried about HPV because if someone has it and they touch you it will spread”
		General Prevention	This subtheme students spoke of prevention/protection in a general and positive way.	“it protects you”
		Cancer Prevention	This subtheme reflects students responses denoting cancer prevention as a positive and motivating factor towards vaccination.	“The most exciting thing to me is that this HPV vaccine could prevent cancer and help build our immune system”
				“The upside is that it helps you not get cancer”
HPV vaccination rates	Student response is related to HPV vaccination rates and their larger impact on society.	Decision to get vaccinated	This subtheme highlights students explicit comments about whether or not they would choose to receive the HPV vaccine. (18 students said they would get it while 6 said they would not get vaccinated)	“I think that everyone should get the shot”
				“I would want to get it”
				“I wouldn’t try it at all and it will kill me”
				“That it will kill the virus. But, I wouldn’t try it.”
		Impact of low HPV Vaccination rates	This subtheme encompasses responses that underscore the relationship between low vaccination rates and persistent spread of HPV.	“If people don’t have a vaccine shot they can have the HPV get spreaded” [*sic*]
				“people aren’t really getting the vaccine”
				“I find that HPV can spread very easily and fast that is why we need HPV vaccine”
				“It spreads to anyone who doesn’t have the vaccine”
		Barriers to HPV Vaccination	This subtheme points towards the structural barriers in place that limit access to the HPV vaccine.	“If your busy and really wanna [*sic*] get a vaccine you won’t have time to get a shot”
				“Everyone may not be able to get one”
		Vaccine advocacy	This subtheme indicates students taking on the role of advocate.	“Parents you need to let your children get a vaccine shot because it’s not bad”
HPV vaccine efficacy	This theme reflects student inquiries and concerns around the effectiveness of the HPV vaccine.			“Will it work?”
				“If you get the vaccine it might not work all the time”
				“The vaccine might not work for that specific type of HPV”
				“The vaccine might not work and I might not be responded to it”
**Knowledge-Related Themes**				
Desired HPV vaccination knowledge	Students displayed a desire for more information related to the HPV vaccine. Subthemes represent the specific types of knowledge desired.	Vaccine administration	This subtheme reflects student’s desire to know more about the vaccine delivery process.	“It will hurt when you get the shot.”
				“Can our parents be in there with us”
				“You have to get a shot! BAD!”
				“The downside is you could get scared because you will be like wonder if its hurts wonder if the needle is sharp”
		Vaccine composition	This subtheme reflects student’s desire to know more about how vaccines are made and what is in the vaccine.	“What is in the shot?”
				“What kind of chemicals are in the vaccine?”
				“What are all the ingredients in the vaccine?”
		Vaccine effects on body	This subtheme indicates students desire to know more about how the vaccine interacts with their body upon administration.	“How long does it take for it to kick in?”
				“I need to know about the vaccine and what it will do to me.”
				“How does it work?”
		Vaccine durability	This subtheme indicates students desire to know more about how long the HPV vaccine will last once the vaccination series is complete.	“We need to know about if it will last forever”
				“You should know if you get the shot how long will it last”
				“Is it going to last for a lifetime or a month?”
Desired HPV knowledge	This theme indicates students desire to gain more information related to HPV overall and not specific to HPV vaccination.			“I want to find out how HPV get in your body and why so many people get it”
				“Why the HPV can make you sick”
				“How does it spread to skin to skin contact”
				“How many people have it and that they can spread it to you”
**Beliefs-Related Themes**				
Perceptions of the HPV vaccine	This theme describes the nature of a student’s overall orientation to the HPV vaccine.	Positive Perceptions of the HPV Vaccine	This subtheme reflects student perceptions that the HPV vaccine is helpful and good for an individual. There was an absence of concern around the HPV vaccine.	“It is helpful”
				“That it is good for you and we need take it”
				“What I find worrisome about the HPV vaccine is that there is nothing to worry about. The vaccine is good for you.”
				“I think it is good to have it because it helps prevent HPV”
				“It’s great and doctors should continue to develop it”
		Negative Perceptions	This subtheme reflects student’s negative perceptions of the HPV vaccine often stemming from vaccine misconceptions and skepticism.	“It is not a good idea to have the vaccine”
				“It is dangerously bad and deadly”
				“I think its just a real virus that’s being injected into you”
				“Some of the vaccines might not work”
